# Consumers' variety‐seeking behaviors under time pressure: Based on regulatory focus and excitement levels

**DOI:** 10.1002/pchj.770

**Published:** 2024-05-15

**Authors:** Yi‐weng Yang, Jia Liu, Yi Wang

**Affiliations:** ^1^ School of Economics and Management North China University of Technology Beijing China; ^2^ Graduate School Central University of Finance and Economics Beijing China; ^3^ School of Business Central University of Finance and Economics Beijing China

**Keywords:** construal level theory, excitement level, regulatory focus, time pressure, variety‐seeking behavior

## Abstract

Variety‐seeking behavior has received substantial attention in marketing literature. Although various explanations of the causes of variety‐seeking explore the influence of consumers' internal psychological characteristics on behavioral decisions, few studies have been conducted on external factors. With the fast pace of modern life and the increasing trend of online shopping, consumers often face time constraints when making purchasing decisions. This study examines the impact of time pressure as a significant external environmental factor on consumers' variety‐seeking behavior. A conceptual framework is developed based on construal level theory to uncover the influencing mechanism of time pressure on variety‐seeking behavior while also considering the effects of the consumer's personality and emotional state. We conducted two experiments to investigate the moderating effect of regulatory focus from the personality perspective and excitement level from the emotional state perspective. Study 1 found that time pressure significantly affects variety‐seeking behavior. Additionally, consumers with prevention regulatory focus tend to exhibit more variety‐seeking behavior when not under time pressure. Study 2 supports the main effect and shows that the level of excitement affects the impact of time pressure on variety‐seeking behavior. Therefore, this study contributes to the literature on consumer behavior and purchasing decisions by presenting a robust theoretical framework that provides practical insights and implications for enterprise managers.

## INTRODUCTION

Time is a crucial resource in an individual's life that is linked to almost every decision they make, including consumption choices. The fast pace of modern life makes time a scarce resource, and time pressure is an unavoidable aspect of our activities, affecting work, leisure, and quality of life (Ackerman & Gross, 2003).

Scholarly interest in this area has significantly increased in recent years. Consumers' tendency to delay decision‐making, cancel shopping, and seek variety under time constraints is well‐documented. However, further empirical evidence is needed to fully understand the factors that drive this behavior. According to Roehm and Roehm's (2004) research, individuals display different levels of variety‐seeking behavior at different times of the day. They noted that people tend to seek more variety during periods of low mental arousal compared to arousal peaks. Fare brands tend to perform better during periods of high variety‐seeking, while leading brands tend to fare better in periods of minimal variety‐seeking. This is because people have varying levels of arousal at different times and require different levels of stimulation for optimal activity. Previous research has confirmed the impact of time perception on variety‐seeking behavior. Therefore, it is important to consider the underlying factors, especially time pressure, to gain a better understanding of the subject.

The current study delves into the effects of time pressure on the spectrum of purchase decisions, with particular focus on the moderating influences exuded by sustained traits such as regulatory focus, which is a new perspective on human motivation in recent years. Regulatory focus theory also emphasizes on how individual behavior approaches the desired end‐states and avoids the undesired end‐states (Higgins, 1997), and transient situational characteristics like excitement levels (Mostafa, 2006).

Building on previous research, we explore the influence of time pressure on consumers' variety‐seeking behavior, while also taking into account the moderating effects of consumer characteristics and emotional states. Our research will aid companies in developing effective marketing, communication, and promotion strategies.

## THEORETICAL BACKGROUND AND HYPOTHESES

### Variety‐seeking behavior

Consumer variety‐seeking behavior is a crucial aspect of purchasing (McAlister, 1982; McAlister & Pessemier, 1982). This behavior is often employed to reduce perceived risk when numerous brands offer little differentiation (Read et al., 1999). According to McAlister and Pessemier (1982), variety‐seeking behavior allows consumers to vary their choices within an acceptable range of options. Consumers can benefit from making alternative choices once they are satisfied with or have grown tired of a particular brand. This is known as variety‐seeking behavior and is apparent when they select particular brands and products (Kahn & Louie, 1991). This tendency is a consumption phenomenon associated with repetitive purchase (Kotler & Armstrong, 2010; Li et al., 2007). In this process, consumers' variety‐seeking behavior is not caused by the brand or product itself, but rather by the functional merit of conversion behavior (i.e., converting into different brands or products; Yu et al., 2008).

Early research on consumer variety‐seeking behavior can be found in the field of psychology, where studies proposed that individuals seek variety to satisfy their need for internal stimuli (Lattin & McAlister, 1985; Menon & Kahn, 1995). People dislike monotony and crave fresh experiences as external stimulation. According to research, product preferences are not the main drive of this behavior (Kahn & Ratner, 2005). Despite having a strong liking for certain items, people still seek variety to increase freshness (Ratner & Kahn, 1999).

Empirical studies have shown that space constraints influence variety‐seeking behavior, with people in crowded spaces more likely to make distinctive choices (Levav & Zhu, 2009). Furthermore, recent studies have explored various aspects of shopping situations, including the relationship between time and variety‐seeking behavior (Chintagunta, 1998; Gullo et al., 2019; Levav & Zhu, 2009; Maimaran & Wheeler, 2008; Roehm & Roehm, 2004; Salisbury & Feinberg, 2012), as well as other factors, such as background music, smell, color, and noise (Beldona et al., 2010; Ratner & Kahn, 2002). Time is a limited resource that influences decision‐making, and consumers make choices within a specific period. Therefore, it is crucial to explore the impact of time pressure on consumers' variety‐seeking behavior.

### Variety‐seeking behavior under time pressure

Time is a scarce resource for modern consumers who often need to make purchasing decisions quickly. According to research in psychology, time pressure is a subjective state in which individuals feel imposed by time constraints, causing emotional reactions, such as anxiety, nervousness, and pain (Mohan et al., 2012; Tuu & Olsen, 2013). Recent studies indicate that time pressure can affect lifestyle, productivity, and consumer purchasing decisions (Afonso Vieira & Vaz Torres, 2013). For instance, consumers may engage in variety‐seeking behavior to reduce consumption risks, postpone decision‐making, or forego shopping when pressed for time (Herrington & Capella, 1995; Mohan et al., 2012; Tuu & Olsen, 2013).

Construal level theory (CLT) was derived from temporal construal theory (Liberman & Trope, 1998), and it provides a suitable theoretical framework for elucidating consumer behavior under time pressure. Based on CLT, individuals' interpretation of an event and their abstract psychological representation, as well as their ability to adjust to different time frames, can influence their responses to the event (Trope, 2004; Trope & Liberman, 2003). When consumers are under time pressure, they perceive a closer psychological distance to the time restriction, leading to a stronger sense of urgency (Kim et al., 2020). This sense of urgency may cause consumers to feel that they have limited time to choose from a wide range of products, leading them to prefer familiar alternatives (Campbell & Goodstein, 2001). As a result, consumer variety‐seeking behavior may decrease. Psychological proximity can cause consumers to rely on less explanation when making decisions (Bar‐Anan et al., 2006; Hamilton, 2014; Ledgerwood et al., 2010). This can lead them to primarily consider alternatives that are highly feasible (Liberman & Trope, 1998), which in turn reduces their tendency to seek out variety. Furthermore, consumers use specific criteria to define alternatives (Liberman et al., 2002), which reduces the number of alternatives and consumer variety‐seeking behavior. Additionally, consumers tend to adopt conservative strategies to reduce risks (Keller et al., 2004), such as choosing familiar products, which also reduces their variety‐seeking behavior. Therefore, when time pressure is high, consumers' variety‐seeking behavior decreases. Meanwhile, when there is no time pressure, greater psychological distance leads consumers to make decisions using a high level of explanation. Consequently, consumers exhibit an active consideration of diverse alternatives (Liberman & Trope, 1998), adopt broad and abstract classification criteria for evaluations (Liberman et al., 2002), and tend towards optimistic and positive decision‐making outcomes (Hamilton, 2014; Ledgerwood et al., 2010). Collectively, these factors amplify their variety‐seeking behavior.

Thus, we propose the following hypothesis:

Hypothesis 1 Consumers are more likely to engage in variety‐seeking behavior when there is no time pressure, as opposed to situations with high time pressure.

### Moderating effect of personality: Regulatory focus

Consumers seek variety for two main reasons. First, they do so to reduce perceived risk when numerous brands offer little differentiation (Read et al., 1999). This involves a prevention regulatory focus (Higgins, 1997). After using the same brand or product for an extended period, consumers may develop a sense of boredom and saturation, leading to variety‐seeking behaviors driven by the desire for newness and change (Koschate‐Fischer et al., 2018). This behavior involves a promotion regulatory focus (Higgins, 1997). The regulatory focus theory (Higgins, 1997) explicates the dual dimensions of prevention and promotion regulatory focuses implicated in consumer behavior. This theory forms the cornerstone of our exploration into the moderating role of consumers' regulatory focus on the effect of time pressure on variety‐seeking behaviors.

Regulatory focus is often regarded as a personality trait (Higgins, 2000; Lockwood et al., 2002), and its influence on behavior has been extensively researched (Lanaj et al., 2012). Environmental factors do not typically alter personality, but they can result in different responses to various environmental stimuli. According to Higgins (1997), individuals with a promotion focus adopt an assertive attitude towards many things, emphasizing the significance of improvement, achievement, and potential benefits. Conversely, prevention‐focused individuals tend to exhibit a relatively cautious attitude, emphasizing responsibilities, obligations, and potential individual losses (Higgins, 1997).

When under increasing time pressure, consumers typically perceive an escalation in risk. However, disparate types of consumers exhibit divergent risk preferences. Belli et al. (2024) propose that consumers with a promotion regulatory focus generally display a greater propensity to embrace risk in decision‐making and frequently demonstrate variety‐seeking behaviors. As a result, their variety‐seeking behavior displays less volatility, despite a decline under significant time pressure (Ye et al., 2023). Conversely, prevention‐focused consumers habitually maintain a conservative approach, aiming to circumvent negative outcomes and implementing risk‐aversion strategies. Under the duress of time pressure, their focus on the inherent risks in decision‐making escalates (Higgins, 1997), causing a contraction in their variety‐seeking tendencies. Individuals with a prevention regulatory focus, predictably, perceive potential losses more acutely, exuding a heightened reticence towards decision‐making risk, and a potential bias towards familiar options (Campbell & Goodstein, 2001). These factors contribute to an overall reduction in variety‐seeking behaviors, a tendency that amplifies alongside escalating time pressure.

Therefore, we propose our second hypothesis:

Hypothesis 2 Variety‐seeking behaviors decrease as time pressure increases for consumers with either promotion or prevention regulatory focus. However, the decrease is greater for consumers with the prevention regulatory focus.

### Moderating effect of emotion: Excitement level

Research on consumer variety‐seeking behavior originated in psychology, which proposed that individuals seek variety to satisfy their need for internal stimuli (Lattin & McAlister, 1985; Menon & Kahn, 1995). However, the optimal level of stimulation may vary among individuals. Optimal stimulation level theory explains consumer behavior at different stimulation levels (Steenkamp & Baumgartner, 1992). Therefore, we investigate the consumer's excitement level as a moderating variable.

McAlister's (1982) optimal stimulation level theory suggests that individuals' behaviors are linked to their level of satisfactory stimulation, and that individuals achieve their most satisfactory state at the optimal stimulation level. As previously mentioned, time pressure is a consumer's perception response to the external environment. Consumers engage in variety‐seeking behavior to obtain additional stimuli, which can complicate purchasing decisions. According to environmental theorists, external factors can affect consumers' level of arousal, which in turn influences their purchasing behavior (Mostafa, 2006). Marketing literature suggests that emotional factors significantly influence preferences and decision‐making, in addition to cognitive and environmental factors. Previous studies (Paulhus & Lim, 1994; Ryu & Jang, 2007; Wirtz et al., 2000) have shown that the level of mental arousal, also known as excitement level, which is a basic dimension of emotion, affects social judgments and behaviors, including advertising effects and purchasing decision‐making.

According to Sun et al. (2023), consumers' need for stimulation varies with their excitement levels. When consumers experience high excitement, their intrinsic stimulation levels increase (Gullo et al., 2019), leading to an increased need for external stimuli. To fulfill this need, consumers may engage in stimulation‐seeking behaviors, such as exploratory and approach behaviors (Sun et al., 2023). This behavior has been observed in studies conducted by Etkin & Mogilner (2016). Moreover, consumers with high mental arousal levels exhibit more variety‐seeking behaviors compared to those with low arousal levels when time pressure is low. However, when time pressure is high, the pressure itself largely satisfies the need for stimulation for consumers with high arousal levels. Furthermore, consumers do not need to seek additional variety to satisfy their need for stimulation. Therefore, we expect that high‐arousal consumers will exhibit a more rapid decline in variety‐seeking behavior as time pressure increases, while low‐arousal consumers will exhibit less pronounced changes.

Thus, we propose our final hypothesis:

Hypothesis 3 Consumers with high and low levels of excitement experience a decrease in variety‐seeking behavior as time pressure increases. However, those with the high level of excitement experience a greater decrease in such behavior.

## STUDY 1

### Objective

Study 1 investigates our hypotheses, explores the impact of time pressure (i.e., high vs. no time pressure) on variety‐seeking behavior among consumers to confirm Hypothesis 1. Furthermore, we examine the moderating effect of regulatory focus on the main effect in the experiment to confirm Hypothesis 2.

### Procedures

#### Pre‐experiment

To determine the amount of time required to create time pressure, we conducted a pre‐experiment involving 30 randomly selected student participants. They completed two tasks, and we recorded the number of seconds it took them to complete both tasks. The Ethics Review Board of the Business School of Central University of Finance and Economics approved our study, and every participant signed an informed consent form.

Task 1 was a reading assignment that required participants to read the story “Uncovering Foxconn: 4:00 AM in the Factory” and answer four related questions. For Task 2, participants were asked to select 10 bottles of juice from supermarket shelves based on their preferences. The data were analyzed using SPSS 20.0, and it was found that the time required for participants to complete both tasks followed a normal distribution. After conducting interviews with experts and having discussions with the research team, it was concluded that the time provided caused high time pressure, resulting in 75% of subjects being unable to complete the task. To measure time pressure in the experiment, we used the 1st quartile (Q1) of the time taken for the two tasks.

In a time‐pressured setting, subjects may be unable to choose as many products as they desire due to a lack of time rather than a lack of desire to seek diversity. To prevent this situation, we asked a *yes*/*no* question: “Did you have to choose fewer products due to lack of time?” If more than 25% of the subjects answered *yes*, we extended the task time by 5 s and re‐ran the pre‐experiment with different subjects until more than 75% of the subjects answered *no*. Descriptive statistics showed that the time required to induce a perception of time pressure was 109.25 s for Task 1 and 62.75 s for Task 2.

#### Formal experiment

Data were collected from 278 college students (130 males and 148 females) at the Central University of Finance and Economics who volunteered to participate in this experiment. None of the participants had previously taken part in similar studies.

The subjects were randomized into two groups (high/no time pressure) with time pressure and variety‐seeking behavior as the independent and dependent variables, respectively, and regulatory focus acting as a moderator. The experiment was conducted entirely online, using countdown tools provided by the questionnaire system. To ensure data accuracy, all steps were completed in a laboratory.

The experiment consisted of three steps (see Appendix A). In Step 1, participants completed a reading task designed to create time pressure. Participants in the high‐time‐pressure group were given 110 s to complete the task, based on the pre‐experiment results of 109.25 s. Participants in the no‐time‐pressure group had no time limit. Each participant read a story and answered four questions related to its content.

In Step 2, participants completed the adjusted Regulatory Focus Questionnaire (RFQ) after the regulatory focus manipulation (see Appendix B). Finally, in Step 3, participants completed the beverage‐purchasing task (see Appendix C) and the time‐pressure scale. Participants in the high‐time‐pressure group were given 63 s to complete the task, based on the 62.75 s obtained in the pre‐experiment, while those in the no‐time‐pressure group had no time limit. Additionally, demographic information and affect indexes were collected in this step.

Variety‐seeking behavior was measured by the number of different types of products purchased by the participants, rather than the number of individual products purchased (Argo et al., 2005; Yi et al., 2020). To eliminate the potential impact of emotional response resulting from exposure to the reading material, participants were asked to rate their feelings on seven‐point semantic differential scales (see Appendix D), which were later combined to create an affect index.

### Materials

#### Reading materials

For this experiment, two different reading materials were selected to manipulate time pressure for two groups. The high‐time‐pressure group read “Uncovering Foxconn: 4:00 AM in the Factory,” while the second group, with no time pressure, read “Slow‐Tempo Life, I Like.” Additionally, participants had to answer four questions based on the reading material, indicating their understanding of the article's content by choosing whether the statements were true or false.

The article titled “Uncovering Foxconn: 4:00 AM in the Factory,” read by the first group, is a 1400‐word news piece that describes the working conditions of Foxconn employees in their “dormitory–workshop–restaurant” routine. The employees are required to complete an installation task on the workshop's assembly line within a fixed amount of time, leaving them with limited leisure time after work. The pre‐experiment revealed that all the subjects who finished reading this article felt tremendous time pressure.

The second group read a 1340‐word blog post titled “Slow‐Tempo Life, I Like.” The blog describes a period of the author's life during a vacation when she did not have to wake up early or see clients. The author was able to sleep until she naturally woke up and had enough time to engage in activities such as drinking tea, yoga, and reading. The author felt relaxed, comfortable, and joyful during this time. The pre‐experiment showed that the participants who completed reading the material experienced feelings of relaxation and pleasure, without any sense of time pressure.

#### Beverage‐purchasing task

Menon and Kahn (1995) propose that beverage purchasing is an ideal experiment for measuring variety‐seeking behavior. According to the dissimilarities, riskiness, and familiarity ratings of different beverages, fruit juices are better suited to measuring variety‐seeking behavior than colas and lemon‐lime sodas. After visiting several supermarkets, we selected Minute Maid, a popular juice brand that offers five flavors (see Appendix C) for the experiment. The guidelines for the task are identical to those described in Section “Pre‐experiment” for the pre‐experiment.

To account for the potential influence of familiarity and preference on the results, participants were asked the following questions: (1) “Do you frequently purchase juice? If the answer is *no*, the next two questions can be skipped.” (2) “Are you familiar with the Minute Maid brand of juice?” (3) “Please list the three most commonly purchased brands of juice.” Invalid questionnaire results from participants who did not frequently purchase juice and who were completely unfamiliar with this brand were excluded.

#### Time‐pressure scale

A time‐pressure scale was used to measure the subjects' perceived time pressure. Participants rated their perceived time pressure on a scale of 1 to 10, with 1 indicating *no pressure* and 10 indicating *significant pressure*. The following questions were asked. (1) The available time appears to be insufficient. (2) I am unable to allocate sufficient time to read the article. (3) I am unable to dedicate enough time to carefully consider each issue. (4) I am confident that I answered all the questions correctly in Task 1. (5) I am highly satisfied with my choice in Task 2.

#### Adjusted RFQ

The RFQ developed by Higgins & Spiegel (2004) was initially used to measure the type of regulatory focus for each participant. The questionnaire consisted of 11 questions, six of which measured promotion focus and the remaining five assessed prevention focus.

To account for potential cultural differences in the effectiveness of the RFQ, we utilized an adjusted RFQ scale developed by Yao et al. (2008), which is better suited for measuring regulatory focus among Chinese consumers. The adjusted questionnaire reduces the number of questions related to promotion focus from the original RFQ, resulting in a total of 10 questions, with four and six questions measuring promotion and prevention focus, respectively (see Appendix B).

### Results

#### Manipulation check

To test the effectiveness of manipulating time limits, we assessed differences in participants' perceived time pressure in Study 1.

Participants rated their sense of time pressure on a scale from 1 to 10, with a lower score indicating little to no time pressure and a score of 10 indicating the highest level of time pressure.

**FIGURE 1:**
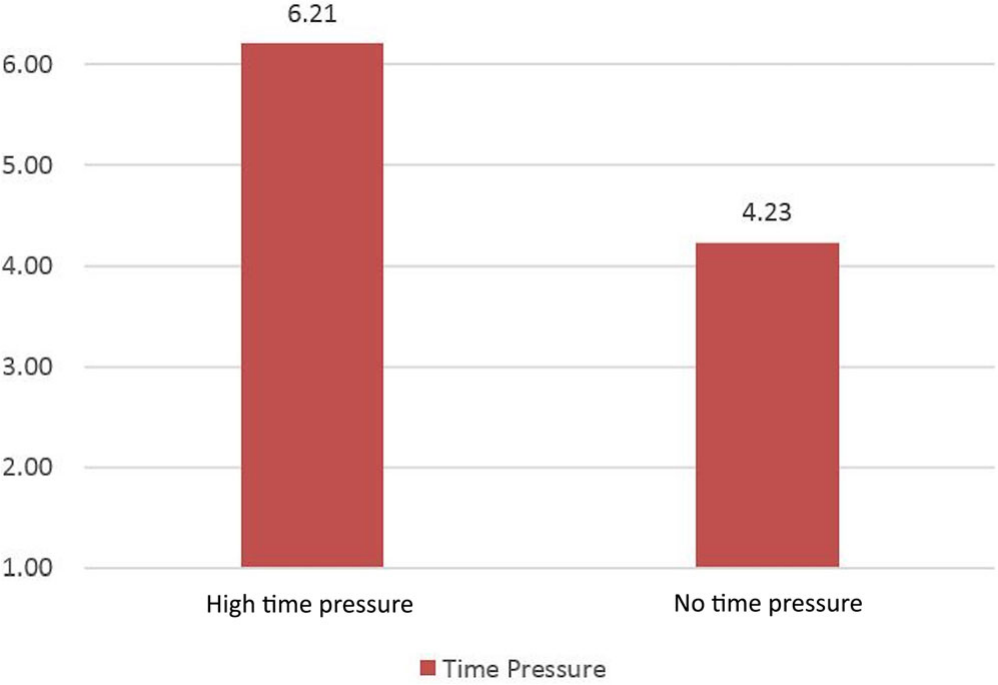
Time pressure perceived by different groups.

The study results clearly demonstrate a significant difference in perceived time pressure between the high‐time‐pressure group and the no‐time‐pressure group (*M*_high time pressure_ = 6.21, *M*_no time pressure_ = 4.23, *p* < .010), providing strong evidence that our time‐pressure manipulation was effective (refer to Figure 1). Participants in the high‐time‐pressure group reported feeling considerably stronger time pressure, while those in the no‐time‐pressure group reported feeling significantly less time pressure. We can confidently state that the affect factor was not a relevant variable in this experiment, as we found no significant differences between the affect index of the two groups after reading the story (*t* = 1.417, *p* = .326).

#### Main effect testing

**FIGURE 2:**
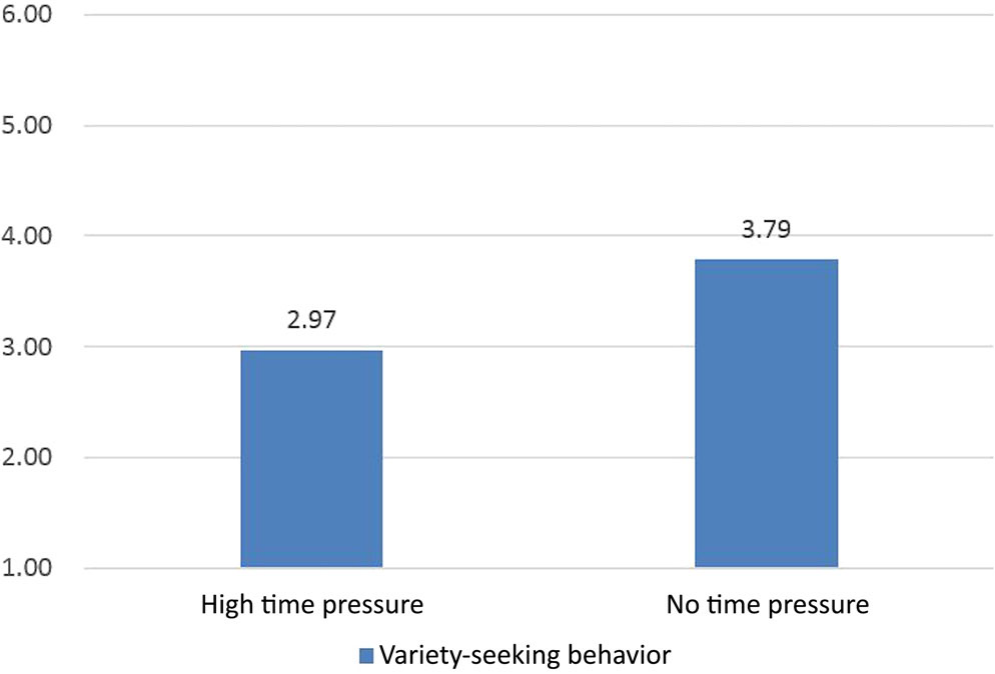
Variety‐seeking behavior under different time pressures.

The study conducted a variance analysis on the levels of variety‐seeking behavior of two groups using SPSS 20.0. The results showed that the average levels of variety‐seeking behavior for the high‐ and no‐time‐pressure groups were *M*_high time pressure_ = 2.97 and *M*_no time pressure_ = 3.79, respectively (refer to Figure 2). The analysis of variance (ANOVA) results indicate a significant difference in variety‐seeking behavior levels between the high‐ and no‐time‐pressure groups (*p* = .026). Specifically, the high‐time‐pressure group exhibits a lower level of variety‐seeking behavior.

#### Regulatory focus as a personality moderator

Regulatory focus significantly impacts the influence of time pressure on seeking variety‐related behavior. The questionnaire survey conducted after manipulation successfully measured the participants' regulatory focus type. The ANOVA results clearly indicate that the promotion‐focus group scored significantly higher on the promotion‐focus items than the prevention group (*M*_promotion focus_ = 2.73 and *M*_prevention focus_ = 1.79, *p* < .010); therefore, we conclude that our regulatory focus manipulation was successful.

We compare the high‐ and no‐time‐pressure groups and present the results in Table 1. The effects of time pressure and the interaction term on variety‐seeking behavior are significant.

**TABLE 1:** Analysis of variance results.

Variable	*df*	*F*
Time pressure	1	15.67 (*p* < .01)
Regulatory focus	1	0.7 (*p* > .05)
Time pressure × Regulatory focus	1	20.59 (*p* < .01)

The hypothesis that variety‐seeking behaviors decrease as time pressure increases for consumers with a promotion and prevention regulatory focus was verified through a series of variance analyses. It was found that the decrease in variety‐seeking behaviors is greater for consumers with the prevention regulatory focus, even after controlling for age, gender, and affect.

**FIGURE 3:**
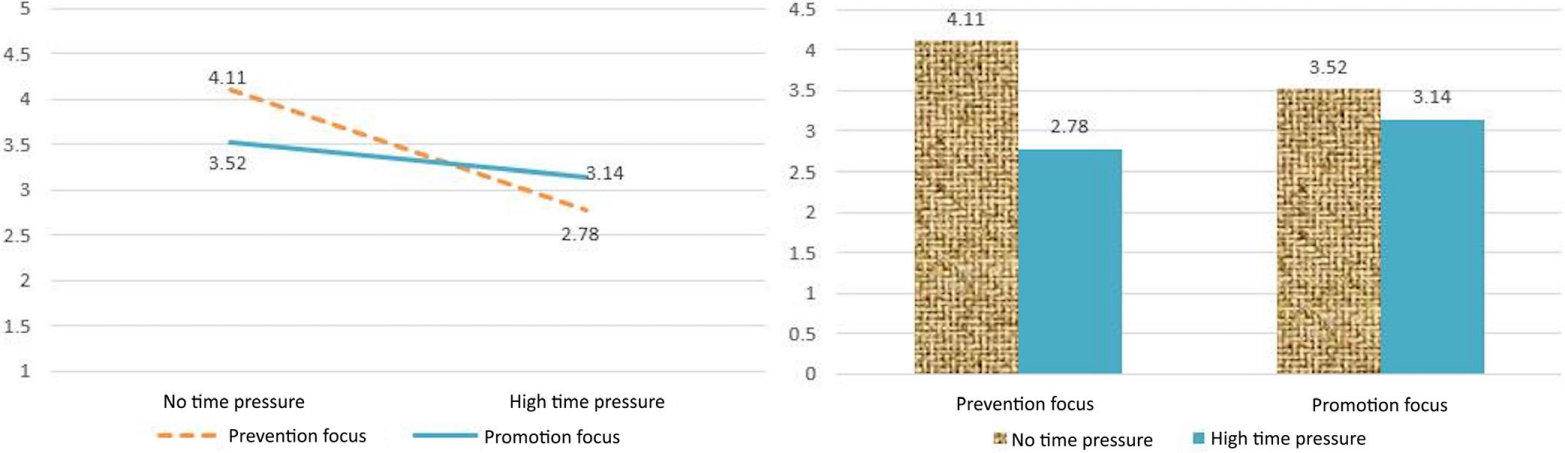
Impact of time pressure and regulatory focus on variety‐seeking behavior.

The variety‐seeking behavior of the prevention‐focus group under varied time pressure conditions was analyzed using an ANOVA. Individuals demonstrated greater variety‐seeking behaviors when they had no time pressure (*M* = 4.11) compared to when they had high time pressure (*M* = 2.78), indicating a significant difference (*p* = .013). These findings suggest a clear relationship between time pressure and variety‐seeking behaviors, with individuals performing better when they have more time. Additionally, those with a prevention focus exhibited significantly more variety‐seeking behaviors under no time pressure compared to high time pressure (*F* = 9.133, *p* = .007; refer to Figure 3).

The ANOVA conducted on the variety‐seeking behavior of the promotion‐focus group under different time pressures revealed no significant variation in variety‐seeking behaviors under high or no time pressure, with an average of *M*_high time pressure_ = 3.14 and *M*_no time pressure_ = 3.52 and a *p* = .438 (refer to Figure 3).

#### Conclusion

The study's main objective is to examine the impact of time pressure on variety‐seeking behavior, specifically comparing high versus no time pressure, while also taking into account the moderating effect of regulatory focus.

The analysis confirms Hypotheses 1 and 2, with Figure 3 summarizing the results and indicating that individuals are more likely to exhibit variety‐seeking behaviors in situations with no time pressure compared to high time pressure. Individuals with a prevention‐focused regulatory style exhibit significantly greater levels of variety‐seeking behaviors in situations without time pressure, compared to those with a promotion‐focused regulatory style.

These results suggest that the influence of time pressure on variety‐seeking behaviors varies depending on an individual's regulatory focus. Time pressure significantly impacted the variety‐seeking behaviors of prevention‐focused individuals, while having little effect on the behavior of promotion‐focused individuals.

## STUDY 2

### Objective

Study 2 aims to determine the impact of excitement level as a moderator, testing both Hypothesis 3 and the main effect. Additionally, this study investigates the potential interaction effect of time pressure and excitement level to determine whether a relationship exists between them.

### Procedures

We recruited 235 college students from the Central University of Finance and Economics who volunteered to participate in our experiment. Participants received a cash reward of 10.00 CNY upon completion. Prior to the experiment, we communicated with the participants and utilized the reward system to enhance their motivation and increase efficiency. Our study was approved by the Ethics Review Board of the Business School at the Central University of Finance and Economics, and all participants provided informed consent.

To examine the impact of excitement level on the main effect, we incorporated a moderation variable and conducted a 2 (high/low time pressure) × 2 (excited/bored) experiment. The excitement level was manipulated by showing an interesting talk show program to the participants in Classroom A (excited group), while those in Classroom B (bored group) watched a solemn and tedious video. After viewing the video, participants in each classroom were divided into two groups, A1 and A2, B1 and B2, respectively. Then participants were asked to complete Questionnaire A1, A2, B1, and B2. Questionnaires A1 and A2 were for the “excited group,” while B1 and B2 were given to the “bored group.” Questionnaires A1 and B1 were designed to elicit high time pressure, while A2 and B2 were intended to elicit no time pressure, and thus achieved the implementation of a 2 × 2 experimental design. Each group completed a different set of questionnaires. Please refer to Appendix E for details on the experiment design and questionnaires.

In Step 1, participants in the two classrooms watched different videos, and then groups A1 and A2 (Classroom A, excited) were asked to recall interesting situations from the video they had watched and rate the level of excitement they had experienced during those times. Additionally, the B1 and B2 groups (Classroom B, bored) were asked about their attitudes toward the video they had watched, with the aim of inducing boredom, and to rate their level of excitement.

In Step 2, participants in the different time‐pressure manipulations (high time pressure: A1 and B1; no time pressure: A2 and B2) were asked to read a short article and answer questions related to keywords of time pressure. For Groups A1 and B1, participants read the article entitled “Life in a Hurry,” and then identified the key words in the article with guidance direction of high time pressure; while for Groups A2 and B2, the reading material was entitling “Lazy Time,” and participants then identified the key words in the article with guidance direction of no time pressure. After that, participants were asked to complete the time‐pressure scale.

In Step 3, participants accomplished the chocolate‐purchasing task (see Appendix F) under the same length of time control as in Study 1. Participants in the high‐time‐pressure group (A1 and B1) were given 63 s to complete the task, while those in the no‐time‐pressure group (A2 and B2) had no time limit. Additionally, we asked all participants about the experiment's purpose to minimize the social desirability bias, that is, systematic error in self‐report measures resulting from the desire of respondents to avoid embarrassment and project a favorable image to others (Fisher, 1993). We found that most participants could not answer correctly for the purpose of the experiment, so the social desirability bias was not severe (Larson, 2019). Finally, we collected demographic information and affect index.

### Materials

This experiment examines the effect of time pressure on consumer variety‐seeking behavior using chocolate as the experimental material. To avoid duplicating Study 1, a virtual brand (Join Joyful) was presented to mitigate the effects of brand familiarity. Appendix F presents the 10 flavors of the virtual brand. Participants were informed that the purpose of the experiment was to conduct market research for a new chocolate company.

To stimulate different levels of excitement among the participants, we used two videos. For the excited group, we used a popular crosstalk program called “Full of Laugh.” For the bored group, we used a video about the death of a highly regarded Prime Minister titled “Farewell to Prime Minister Zhou on CHANG‐AN Street.” The method used to measure variety‐seeking behavior is based on previous studies (Argo et al., 2005; Yi et al., 2020), which is consistent with Study 1.

### Results

#### Manipulation check of excitement level

In Study 2, we manipulated the level of excitement by showing two different videos.

One video was an interesting talk show that was designed to increase participants' excitement level, while the other was meant to be depressing. To assess whether the subjects' level of excitement corresponded to our expectations, they were asked to recall the most interesting or boring part of the video they viewed and rate their level of excitement on a 7‐point scale, ranging from *bored* to *excited*.

**FIGURE 4:**
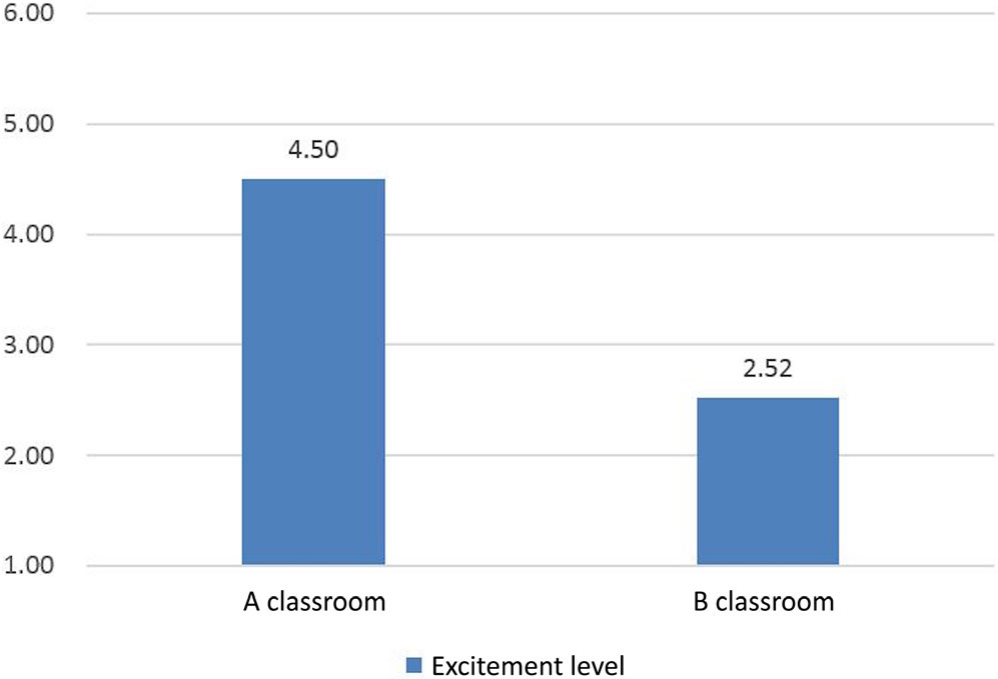
Excitement levels in Classrooms A and B (manipulation check).

After analyzing 230 valid questionnaires, we determined that the average excitement level of participants who watched the crosstalk video (129 students in Classroom A) was 4.50, compared to 2.52 for those who watched the sadder video (101 students in Classroom B). Figure 4 displays these results. An ANOVA revealed significant differences in the excitement level of subjects in the two classrooms (*t* = 8.596, *p* < .010). Thus, we conclude that the experiment effectively manipulated the participants' level of excitement by presenting them with two distinct videos.

#### Time‐pressure analysis

The method employed to evaluate time pressure in Study 2 is identical to that of Study 1. To confirm the intended impact of the time‐pressure manipulation, participants were asked in the initial section of the questionnaire to rate on a 7‐point scale whether they experienced any time pressure after reading the two stories. The scale ranged from 1 (*no pressure at all*) to 7 (*extremely high pressure*) to eliminate the scale's influence, unlike the 10‐point scale used in Study 1.

**FIGURE 5:**
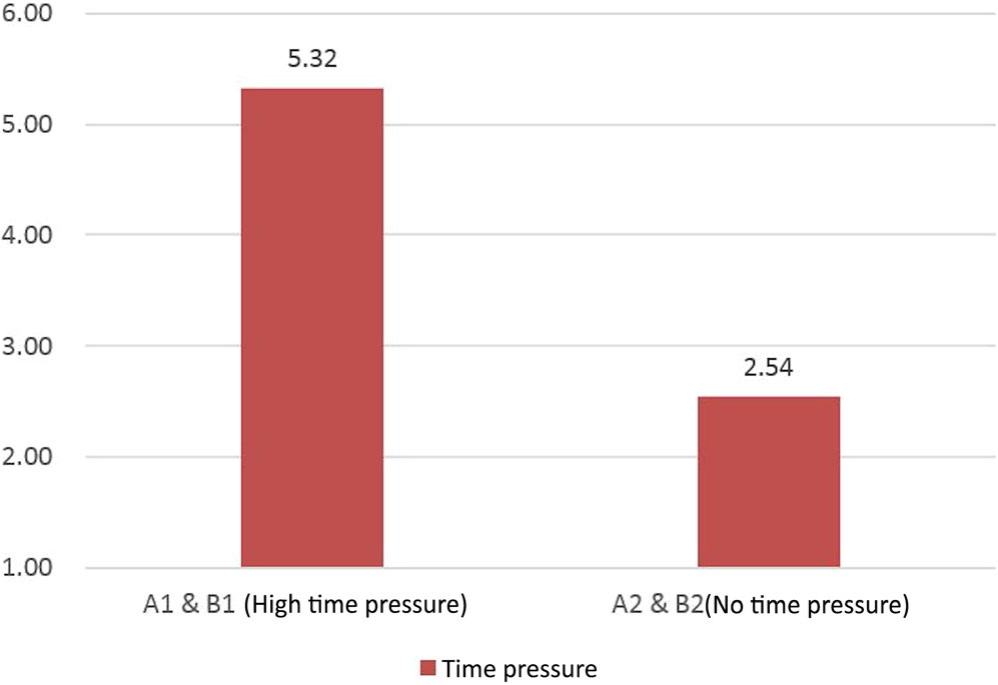
Time pressure in different groups (manipulation check).

In Classroom A, 66 participants were randomly assigned to the high‐time‐pressure group (A1) and 63 to the no‐time‐pressure group (A2), out of a total of 129 participants. Similarly, in Classroom B, 50 students were assigned to the high‐time‐pressure group and 51 to the no‐time‐pressure group. The mean scores of the high‐ and no‐time‐pressure groups (A1 and B1 vs. A2 and B2) were compared, revealing an average score of 5.32 for the high‐time‐pressure group and 2.54 for the no‐time‐pressure group (Figure 5). The ANOVA showed significant differences in perceived time pressure between the two groups (*t* = 7.342, *p* < .010), indicating successful manipulation of time pressure during the reading task.

#### Variety‐seeking behavior

Study 2 examines the impact of time pressure on variety‐seeking behavior, while considering the moderating effect of excitement level.

The dependent variable in this study is variety‐seeking behavior. We used various statistical analyses, including correlation analysis, ANOVA, and regression analysis, to analyze the data obtained from our experiments.

A correlation analysis was conducted to investigate the relationship between variables, namely time pressure, excitement level, and variety‐seeking behavior. The results, presented in Table 2, indicate a significant negative correlation between time pressure and variety‐seeking behavior in Study 2 (*p* < .05). Additionally, the correlation between excitement level and variety‐seeking behavior is significant at the 10% level (Table 1, *p* < .100). No significant correlation was found between excitement level and time pressure.

**TABLE 2:** Descriptive statistics and variable correlations.

Variable	*M*	*SD*	1	2	3
1. Time pressure	3.96	1.86	1	‐	‐
2. Excitement level	3.50	1.64	0.048*	1	‐
3. Variety‐seeking behavior	3.72	1.93	−0.580**	0.060	1

* *p* < .10.

** *p* < .05.

The regression analysis indicates that time pressure significantly affects variety‐seeking behavior, even when the excitement level variable is not included, thus supporting Hypothesis 1. Additionally, excitement level itself significantly influences variety‐seeking behavior (refer to Table 3). However, upon adding the interaction term between excitement level and time pressure, we observe no impact on variety‐seeking behavior; it does indeed interfere with the effect of time pressure on variety‐seeking behavior.

**TABLE 3:** Regression analysis results.

	Model 1			Model 2			Model 3		
	*B*	*t*	Sig	*B*	*t*	Sig	*B*	*t*	Sig
TP	−0.602	−8.030	.001	‐	‐	‐	−0.390	−2.292	.024
EL	‐	‐	‐	0.071	0.675	.051	0.346	1.803	.074
TP × EL	‐	‐	‐		‐	‐	−0.063	−1.410	.161
*F*	64.474	‐	‐	2.456	‐	‐	22.897	‐	‐
Sig	.001	‐	‐	.051	‐	‐	.001	‐	‐
△*R* ^2^	0.580	‐	‐	0.060	‐	‐	0.596	‐	‐

Abbreviations: EL, excitement level; TP, time pressure.

#### Conclusion

Study 2 investigates whether time pressure affects variety‐seeking behavior and explores the potential interference effect of excitement level and time pressure.

**FIGURE 6:**
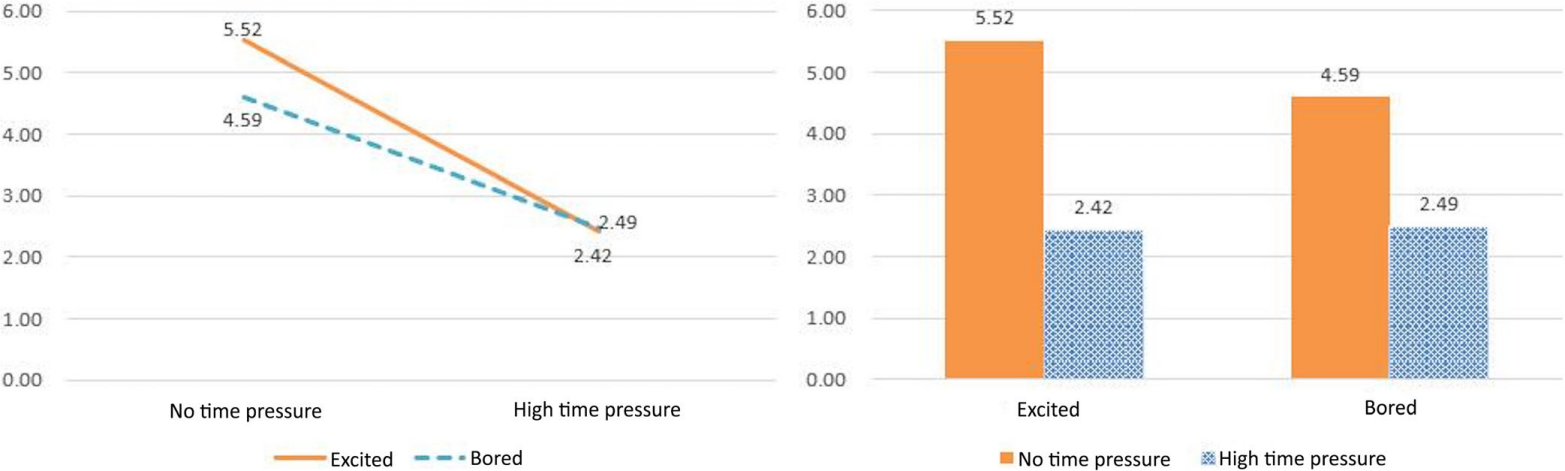
Variety‐seeking behavior under different time pressures and excitement levels.

The results support Hypothesis 1 and partially support Hypothesis 3. The findings indicate that consumers tend to decrease their variety‐seeking behavior under higher time pressure, and excitement level can moderately interfere with the effects of time pressure on variety‐seeking behavior. However, the level of excitement does not directly impact variety‐seeking behavior. Figure 6 illustrates the differences in variety‐seeking behavior under high versus no time pressure and high versus low excitement levels.

These results suggest that the level of excitement plays a moderating role in the relationship between time pressure and consumers' variety‐seeking behavior, which is consistent with our main hypothesis. Furthermore, our findings indicate that the level of excitement interacts with time pressure but does not directly impact variety‐seeking behavior. We used correlation and regression analyses to examine the results of Study 2, and we employed a different analysis method to avoid duplicating Study 1 and to eliminate possible interference from the methods.

## GENERAL DISCUSSION

This study offers an in‐depth analysis of the influence of time pressure on variety‐seeking behavior among young consumers and explores the moderating effects of regulatory focus and excitement level. The data were collected from college students to verify our hypotheses.

The results of the study support Hypothesis 1, showing that time pressure significantly influences variety‐seeking behavior. We show that consumers tend to engage in variety‐seeking behavior more often when they feel no time pressure compared to situations with considerable time pressure. This finding confirms to CLT, predicting that in the absence of time pressure, individuals tend to display high‐level construal, leading to increased variety‐seeking behaviors while shopping. Conversely, under high time pressure, individuals focus more on their immediate needs and tend to display low‐level construal, decreasing variety‐seeking behavior by opting for familiar products.

Our results also support Hypothesis 2, showing that in the absence of time pressure, individuals with prevention focus exhibit significantly more variety‐seeking behaviors than those under high time pressure. However, for consumers with the promotion focus, time pressure does not significantly affect variety‐seeking behavior. Further exploration is necessary to understand the underlying factors that affect variety‐seeking behavior of consumers that are not considered in our experimental design. Additional studies conducted under strict laboratory conditions may be needed to verify this conjecture.

Our findings only partly support Hypothesis 3, as we find young consumers' excitement levels interfere with the impact of time pressure on variety‐seeking behaviors. Moreover, we find that excitement level moderates the relationship between time pressure and variety‐seeking behavior but does not have a direct effect on variety‐seeking behavior.

### Theoretical contributions

In contemporary societies, the pace of life is accelerating, and consumers frequently need to make consumption decisions quickly. At the same time, consumers often face time constraints, such as “limited time only,” “sold out,” and “limited quantity”. Therefore, researching how consumers make decisions under time pressure has become crucial. Several studies have investigated the impact of time pressure on consumer behavior, including choice deferral (Dhar & Nowlis, 1999), choice utility (Godinho et al., 2016), and browsing behavior on shopping websites (Liu et al., 2017), among other factors.

However, consumer seeking behaviors are often diverse and driven by the desire for novelty and change. Consumer variety‐seeking behavior can be an effective market segmentation criterion that businesses can use to increase sales and market share. Studies have shown that this behavior is influenced by both internal and external factors. Internal factors include demographics, personality characteristics, emotions, physical state, sensory clues, and mindset. External factors include the social environment, physical environment, and marketing strategy (Zhang, 2022).

This study contributes to the existing literature by establishing a link between time pressure and consumer variety‐seeking behavior. It is important to note that time pressure is an external factor that significantly affects consumer decision‐making. This study is the first to establish this link, which is a significant contribution to the field. This study explores the impact of time pressure on consumers' variety‐seeking behavior based on the CLT. The findings indicate that when time pressure is high, consumers' variety‐seeking behavior tends to decrease, which supports the hypothesis. However, it is worth noting that not all individuals feel that “hungry is no choice, panic is no choice,” and consumers who exhibit regulation of promotion focusing do not alter their variety‐seeking behavior under different time pressures. These findings enhance our understanding of consumer behavior under time pressure, enrich research on the effects of time pressure on consumer decision‐making and factors influencing consumers' variety‐seeking behaviors, and extend the application of the CLT.

Second, based on regulatory focus theory, limited studies have explored the direct effects of regulatory focus on consumers' variety‐seeking behavior. For instance, Cui et al. (2023) found that consumers with a prevention regulatory focus exhibit less variety‐seeking behavior toward food than consumers with a promotion regulatory focus. Regulatory focus is an important personality trait that is often used as a moderating variable to examine the effects of individual psychological and behavioral antecedents on outcome variables (Higgins et al., 1997). However, no studies have examined the moderating effect of regulatory focus on the influence of antecedent variables on consumers' variety‐seeking behavior. This study confirms that regulatory focus plays a moderating role between the effects of time pressure on consumers' variety‐seeking behavior, based on the theory of regulatory focus. Therefore, our findings advance the literature on the application of regulatory focus theory to consumers' variety‐seeking behavior.

Third, the optimal arousal level theory has been used to investigate variety‐seeking behavior in consumption (Zhang, 2022). Studies have shown that when consumers have a low arousal level, they tend to purchase different products to increase their arousal level (Roehm & Roehm, 2005). However, no study has examined the moderating effect of arousal level on the antecedent variables of consumers' variety‐seeking behavior based on the optimal arousal level theory. This study confirms that the level of arousal moderates the effect of time pressure on consumer variety‐seeking behavior. This finding contributes to the study of optimal arousal theory as it applies to consumer behavior.

### Managerial implications

This study provides insights for practitioners regarding the relationship between time pressure and consumers' variety‐seeking behavior. First, the findings suggest that time pressure decreases variety‐seeking behavior, while the absence of time pressure increases it. To control variety‐seeking behavior, companies can apply different levels of time pressure on consumers in different situations. For instance, to increase consumer loyalty, companies could use strategies such as “limited time offer,” “sold out,” or “limited quantity” to reduce variety‐seeking behavior. To attract customers and promote product exploration, companies can create a stress‐free shopping experience by implementing strategies such as a “15‐day price guarantee,” “lock‐in first,” “7‐day no‐excuses returns,” and “order cancellation at any time”.

Second, we show that consumers with a prevention regulatory focus are more susceptible to time pressure when engaging in variety‐seeking behaviors than those with a promotion regulatory focus. In the long term, companies can identify consumers with a stable prevention regulatory focus through big data analyses, artificial intelligence, and other technologies. They can then use strategies, such as those described earlier, to control those consumers' variety‐seeking behavior by applying time pressure. Meanwhile, in the short term, companies can manipulate consumers' situational regulatory focus (Higgins et al., 2001) to increase variety‐seeking behavior. To awaken consumers' sense of duty and responsibility when time pressure is low, companies can employ strategies that stimulate consumers' preventive regulatory focus. Conversely, when time pressure is high, companies can use strategies that awaken consumers' ideals, hopes, and aspirations to stimulate their promotion regulatory focus (Pham & Avnet, 2004). Furthermore, to reduce consumers' tendency to seek variety, companies should aim to promote a regulatory focus when time pressure is low and a preventive focus when time pressure is high.

Third, it has been determined that when time pressure is low, consumers with higher levels of arousal display more variety‐seeking behaviors than those with lower arousal levels. However, when time pressure is high, there is little difference in variety‐seeking behaviors, regardless of arousal level. Therefore, companies that want to increase variety‐seeking behaviors when time pressure is low can use various strategies to increase consumers' arousal levels. Furthermore, individuals have varying levels of arousal throughout the day, which is dependent on their individual chronotype (Gullo et al., 2019). To increase variety‐seeking behavior, it may be more effective to target “lark” consumers in the morning and “night owl” consumers in the evening.

### Limitations and future research

Although this study provides significant theoretical and practical insights, there are still limitations and areas that require improvement.

The optimal stimulation level model (McAlister & Pessemier, 1982) is a fundamental theoretical model that identifies intrinsic motivation in consumers' variety‐seeking behavior. However, recent studies have not measured this variable, and there is no mature scale available for it. Developing a scale to measure optimal stimulation level would enhance future studies in this area.

Our study used only university students as subjects for our experiments. Although we minimized the potential influence of demographic variables, a more diverse sample would be beneficial. The university student cohort cannot represent all consumer characteristics, which limits the external validity of our findings. To enhance the generalizability of our study, future research should broaden the participant pool and diversify variables such as age and occupation to create a more representative sample, thereby improving the applicability of our findings.

Additionally, consumer heterogeneity encompasses social task orientation, self‐control, brand preferences, and other factors that could influence characteristics and excitement levels, resulting in varying impacts on consumers' decision‐making frameworks. Therefore, additional research is necessary to verify the potential moderating effect on variety‐seeking behavior. Furthermore, future studies could use alternative research designs and experimental approaches to validate our study's findings.

## AUTHOR CONTRIBUTIONS

All authors conceptualized the manuscript. JL and YW wrote the first complete draft. YY contributed additional writing and further analysis, JL, YW, and YY contributed data collection and analysis, and all authors edited the manuscript and approved the final version. All of the authors contributed equally to this work as co‐first authors.

## FUNDING INFORMATION

The current work was supported by the National Natural Science Fund of China (NSFC) awarded to Yi Wang (71472192), National Social Science Foundation (NSF) to Yi Wang (23ZD15), the Humanities and Social Science Fund of Ministry of Education of China awarded to Yi‐weng Yang (22YJC630186), National Social Science Foundation (NSF) (23BGL144), the Project of Cultivation for Young Top‐Notch Talents of Beijing Municipal Institutions awarded to Yi‐weng Yang (BPHR202203038), and “the Fundamental Research Funds for the Central Universities”.

## CONFLICT OF INTEREST STATEMENT

The authors declare that they have no known competing financial interests or personal relationships that could have appeared to influence the work reported in this paper.

## ETHICS STATEMENT

The Ethics Review Board of the School of Business in Central University of Finance and Economics approved our study, and every participant signed an informed consent form. Therefore, there is no ethics risk in our study. This article was created by the three named authors and does not involve plagiarizing researches of others.

## APPENDIX A Design of the experiment in Study 1

**Table:**

	High time pressure		No time pressure	
Step 1	Read story “Uncovering Foxconn: 4:00 AM in the Factory”		Read story “Slow‐Tempo Life, I Like”	
	Answer simple questions about content of the reading material			
Step 2	Measure time pressure			
	Regulatory focus (Promotion)	Regulatory focus (Prevention)	Regulatory focus (Promotion)	Regulatory focus (Prevention)
	Complete the adjusted Regulatory Focus Questionnaire			
Step 3	Beverage‐purchasing task with high time pressure		Beverage‐purchasing task with no time pressure	
	Demographic information			
	Answer the purpose of this experiment, the affect index of the questionnaire			

## APPENDIX B Regulatory focus manipulation

Task: Briefly list seven recent goals, using keywords and examples.

Promotion Focus

**Keywords:** pursuit, success, gain, achieve.

**Example:** I am eager to be promoted to the supervisor of the department.

Prevention Focus

**Keywords:** avoidance, prevent, mistakes, precaution.

**Example:** I want to avoid forgetting my keys when I go out.

### B.1. The adjusted Regulatory Focus Questionnaire (RFQ) scale

The questionnaire can be reprinted for academic research.

**Table:**

*Promotion focus*	Yao et al. (2008)
Q1: I feel that I have already moved toward success	
Q2: I can perform well for what I want to do	
Q3: Success makes me work harder	
Q4: I want to acquire a lot	
*Prevention focus*	
Q1: I have almost no hobbies in my life	
Q2: I follow the rules set by my parents	
Q3: I do things carefully	
Q4: I often overwhelmed my parents	
Q5: Failure makes me fearful	
Q6: I often annoy my parents	

## APPENDIX D The affect index scale (feelings on seven‐point semantic differential scales)

The questionnaire can be reprinted for academic research.

**Table:**

Four answers subsequently combined to form an affect index	Hagtved (2011)
Q1: *Not at all happy*/V*ery happy*	
Q2: *Not at all excited*/*Very excited*	
Q3: *Not at all hopeful*/*Very hopeful*	
Q4: *In a bad mood*/*In a good mood*	

## APPENDIX E Design of experiment and questionnaires in Study 2

**Table:**

	Classroom A (excited)		Classroom B (bored)	
	A1	A2	B1	B2
	High time pressure	No time pressure	High time pressure	No time pressure
	Questionnaire A1	Questionnaire A2	Questionnaire B1	Questionnaire B2
Step 1	Watch the video “Full of Laughter”		Watch the video “Farewell to Prime Minister Zhou on CHANG‐AN Street”	
	Answer simple questions about content of the video			
	Answer “What do you think is the most interesting part of this video?”		Answer “What do you think is the most boring part of this video?”	
	Measurement of excitation level (7‐point Likert scale)			
Step 2	Reading the article “Life in a Hurry”	Reading the article “Lazy Time”	Reading the article “Life in a Hurry”	Reading the article “Lazy Time”
	Simple question: Identify the key words in the article			
	Guidance direction: time pressure	Guidance direction: no time pressure	Guidance direction: time pressure	Guidance direction: no time pressure
	Measure time pressure (7‐point Likert scale)			
Step 3	Product‐purchase task with high time pressure	Product‐purchase task with no time pressure	Product purchase task with high time pressure	Product purchase task with no time pressure
	Demographic information			
	Answer the purpose of this experiment, the affect index of the questionnaire, and whether you are familiar with chocolate			

Questionnaires A1, A2, B1 and B2

**Table:**

Questionnaire A1 (Excited × High time pressure)	Questionnaire A2 (Excited × No time pressure)	Questionnaire B1 (Bored × High time pressure)	Questionnaire B2 (Bored × No time pressure)
Answer simple questions about content of the video			
Answer “What do you think is the most interesting part of this video?”		Answer “What do you think is the most boring part of this video?”	
Measurement of excitation level (7‐point Likert scale)			
Identify the key words in the article (guidance direction: high time pressure)	Identify the key words in the article (guidance direction: no time pressure)	Identify the key words in the article (guidance direction: high time pressure)	Identify the key words in the article (guidance direction: no time pressure)
Measure time pressure (7‐point Likert scale)			
Product purchase task			
Demographic information			
Answer the purpose of this experiment, the affect index of the questionnaire, and whether you are familiar with chocolate			
